# Association between type D personality and supportive care needs in elderly patients with breast cancer: a prospective longitudinal observational study

**DOI:** 10.1186/s12877-023-04407-x

**Published:** 2023-11-07

**Authors:** Suxing Wang, Yuan Li, Shu Li, Enming Zhang, Zhengyue Dai, Jiasong Cui, Xiaolong Wang, Qiong Fang

**Affiliations:** School of Nursing, Shanghai Jiao Tong University, Shanghai, China

**Keywords:** Supportive care needs, Type D personality, Breast cancer, Elderly

## Abstract

**Background:**

Elderly patients with breast cancer often have more unmet needs after receiving common treatments such as surgery and chemotherapy. Type D personality has been related to supportive care needs in the general population. However, its association with supportive care needs in elderly breast cancer patients has not been prospectively explored. This study aimed to address this gap.

**Objectives:**

The aim was to understand the impact of Type D personality on the supportive care needs of elderly breast cancer patients at diagnosis, 2 weeks postoperatively, 3 months postoperatively, and 6 months postoperatively and to analyse the impact of Type D personality on the changing trajectory of supportive care needs after controlling for confounding factors such as demographics, symptom distress and social support.

**Methods:**

A total of 122 elderly patients (≥ 65 years) with breast cancer in Ruijin Hospital, Shanghai, China, were included from September 2021 until August 2022. Supportive care needs were measured by the Supportive Care Needs Survey Short Form and tracked at diagnosis, 2 weeks postoperatively, 3 months postoperatively, and 6 months postoperatively. To investigate changes in the supportive care needs of elderly breast cancer patients and the effect of Type D personality on these needs, a linear mixed model was applied.

**Results:**

A total of 122 elderly patients participated. There was an overall decreasing trend in supportive care needs, with Type D personality patients having significantly higher levels of supportive care needs than the non-Type D personality patients at all stages. Through linear mixed models, it was found that the Type D personality group had a lower overall downward trend than the non-Type D personality group, with need levels remaining consistently higher. This difference persisted after controlling for demographic information, symptom burden, social support.

**Conclusions:**

Elderly breast cancer patients with Type D personality had higher levels of supportive care needs and a slower rate of decline that was maintained over a longer period than those with non-Type D personality.

## Background

Breast cancer has become the most common cancer worldwide, and according to the latest data from the International Agency for Research on Cancer (IARC), the number of new breast cancer patients worldwide reached 2.26 million in 2020, of which approximately 33.40% were older than 65 years [1]. The ageing trend will further increase the number of elderly breast cancer patients [2]. Breast cancer treatment protocols are usually based on surgery, radiotherapy, and chemotherapy, which are effective in improving survival rates [3]. Nevertheless, these treatments have many side effects, leading to the increased prevalence of severe physical, psychological and social care needs in breast cancer patients [4], and their needs change dynamically with the stage of treatment [5–7]. Meeting the supportive care needs of breast cancer patients at different stages of treatment is one of the most essential safeguards for improving their quality of life and health outcomes [8, 9].

Supportive care needs refer to the help and support cancer patients and their families need to cope with disease-related symptoms and adverse reactions during treatment, to effectively participate in medical decision-making and to reduce the damage caused by the disease and treatment [10]. Meeting patients' supportive care needs can improve their quality of life and reduce their psychological, social and spiritual stress [8, 11, 12]. In contrast, unmet patient needs may lead to ineffective coping, increased emotional distress, and reduced quality of life [13–15]. The Multinational Association for Supportive Care in Cancer (MASCC) advocates that good supportive care can lead to better cancer care [16].

The four actions of the WHO Thirteenth General Program of Work 2019–2023 on promoting healthy ageing also emphasize "providing integrated person-centred care and health care that is responsive to the needs of older people" [17]. Williams [18] surveyed 1460 older people with cancer, including various cancer types such as breast, prostate, and digestive cancers, and showed that approximately two-thirds of participants reported having at least one supportive care need, with the highest needs noted in the subdomains of emotional (49.5%) and physical support (47.4%). Several studies have found that the care of older cancer patients is complex because of the wide variation in health status between individuals and their increased incidence of comorbidities and the potential need for multiple medications [19]. In addition, ageing is usually accompanied by the deterioration of some physiological functions, such as vision and hearing loss, as well as changes in social roles. As a result, older cancer patients often have a higher degree of supportive care needs. In addition, cancer patients' supportive care needs are dynamic. Studies have found that the supportive care needs of cancer patients are not stable but change dynamically according to the stage of treatment [20]. For example, Zhang Xi et al. found that patients' supportive care needs were highest at 2 weeks postoperatively and gradually decreased thereafter [21]. However, recent studies on the supportive care needs of cancer patients have primarily been cross-sectional, with relatively few longitudinal studies, and previous studies do not take into account all associated factors. Tailored care interventions based on the characteristics of cancer patients' supportive care needs at different stages of treatment can effectively meet patients' needs and improve their quality of life while reducing their burden and health care costs [12].

Various factors, including psychological, physiological, and social factors, influence the supportive care needs of cancer patients [22]. Physiological factors such as age, comorbidities, symptom burden, and metastasis all have an impact on patients' supportive care needs [23, 24]. Social support also has a significant influence on the supportive care needs of cancer patients, and it was found that older cancer patients have reduced social support and are more likely to experience problems such as isolation and loneliness, leading to higher levels of supportive care needs [25]. In addition, personality traits, especially Type D personality, should also be considered as one of the influencing factors.

Personality traits can be divided into four types, namely, Type A personality with competitiveness, urgency and a strong desire to achieve, Type B personality with a soothing, submissive and slow pace, Type C personality with repression and depression, and Type D personality with aloofness, avoidance and withdrawal [26]. Type D personality (also known as distress personality) is defined by complex and highly negative emotions plus social inhibition [27]. This personality is associated with increased depressive symptoms [28, 29], leading to a greater tendency to adopt a negative coping style in dealing with disease [28]; therefore, there may be more supportive care needs in the Type D personality group compared to the non-Type D personality group. To our knowledge, the influence of type D personality on supportive care needs is still understudied, and recent studies have primarily been cross-sectional, with relatively few longitudinal studies. No prospective, longitudinal observational studies have been conducted to explore the role of Type D personality in the supportive care needs of older breast cancer patients. Understanding how Type D personality affects the level and trajectory of supportive care needs in elderly breast cancer patients could help clinical staff develop tailored interventions to meet the needs of these patients and help control health care costs. Therefore, this study aimed to explore the relationship between supportive care needs and Type D personality in elderly breast cancer patients and to determine the impact of Type D personality on changes in the supportive care needs of older breast cancer patients through a linear mixed model, controlling for general demographic and disease-related factors, symptom distress, and social support.

## Methods

### Study design and participants

A prospective observational study was conducted. The study was approved by Shanghai Jiao Tong University School of Public Health and Nursing Research Ethics Committee approval (SJUPN-202106), and all methods were performed in accordance with the Declaration of Helsinki. All data were collected anonymously following informed consent, and participation was voluntary. Baseline data (T1) were collected between September 2021 and February 2022 via the consecutive recruitment of patients before surgery in Ruijin Hospital Breast Centre, Shanghai Jiaotong University School of Medicine, China. The participants were requested to fill out a questionnaire fourteen days (T2) after surgery and 3 months (T3) and 6 months (T4) after surgery. The inclusion criteria included the following: (a) patients who were pathologically diagnosed with breast cancer and underwent surgery, (b) those aged 65 years or older and (c) those who provided informed consent. The exclusion criteria were as follows: (a) participants with breast cancer combined with other malignant tumours or failure of vital organs, (b) those with visual, auditory or cognitive disorders(Cognitive dysfunction can be objectively confirmed by history taking or neuropsychological evaluation with at least 2 of the following 5 items: (1) memory and learning impairment; (2) executive functioning such as reasoning, judgment, and processing of complex tasks; (3) visuospatial impairment; (4) impaired language functioning (listening, speaking, reading, and writing); and (5) personality, behavioral, or conduct changes), and (c) those with a mental disability, such as schizophrenia.

### Measures

Demographic and clinical variables were measured. We included demographics (i.e., age, marital status, educational level, primary caregiver), and clinical covariates (i.e., type of surgery, comorbidities, and adjuvant therapy).

Unmet needs were measured using The Chinese version of the Supportive Care Needs Survey Short Form (SCNS-SF34), which was translated and revised by Han [30] according to Boyes’s version [31]; this 34-item self-report questionnaire assesses current psychological (10 items), information (11 items), sexual (3 items), physical and daily living (5 items) and patient care and support (5 items) needs. For each item, participants indicate their level of need over the past month on a five-point scale (1 = no needs, not applicable; 2 = needs satisfied; 3 = low needs; 4 = moderate needs; 5 = high needs). The total scale score is the sum of the five subscale scores, ranging from 34–170. Higher scores indicate greater unmet care needs. The Chinese version has been tested on patients with cancer in China, with a Cronbach’s alpha of 0.947 and a high degree of internal consistency [30].

The Social Support Rating Scale (SSRS) was used in this study to assess the social support of elderly breast cancer patients; this scale was developed by Xiao [32] and consists of three domains including subjective support (Items 1, 3, 4, and 5), objective support (Items 2, 6, and 7) and use of support (Items 8, 9, and 10), with a total of 10 items and scores ranging from 12 to 66. Higher scores indicate higher levels of social support. The scale has a Cronbach's alpha of 0.92, which indicates good reliability [33].

Symptom burden was assessed with the validated MD Anderson Symptom Inventory (MDASI) questionnaire, which was developed by Cleeland and his colleagues [34] to assess symptom burden in patients with cancer. The MDASI consists of two domains containing 19 items. The first part assesses the severity of common symptoms in cancer patients through 13 items, and the second part considers the impact of symptoms on the lives of cancer patients through 6 items. Both domains are scored on a Likert scale from 0–10, with a score of 0 indicating that the symptom is not occurring or that the symptom is not causing distress for the patient and 10 indicating that the symptom has reached its most severe state or that the symptom is causing the most disruption to the patient [34]. The scale is concise and suitable for use with cancer patients in China, with a Cronbach's alpha reliability of 0.8 or above [35].

The Type D Personality Scale was developed by J Denollet [36] and translated by Yu [37]. This scale has been widely used in studies involving cancer patients. The scale identifies Type D personality as being characterized by negative affectivity (NA) and social inhibition (SI) on a 5-point Likert scale. Items are scored on a scale ranging from 0 = false to 4 = true. The higher the score is, the more pronounced the personality trait, and when a patient has an SI score ≥ 10 and an NA score ≥ 10, they can be judged as having a Type D personality. The NA and SI scales are internally consistent (Cronbach's α = 0.88 and 0.86) [28].

Patients' sociodemographic data were collected at baseline, whereas clinical data were extracted from patients' medical records using a standard protocol. The above information was assessed at baseline, and supportive care needs, symptom burden and social support were also evaluated at each subsequent follow-up time point.

### Statistical analyses

We used descriptive statistics to analyse demographic and clinical characteristics. A t test was used to analyse the differences in supportive care needs between the Type D personality group and the non-Type D personality group at each time point. To analyse the effects of Type D personality (between subjects) and the interaction term between Type D personality and time (within subjects) on supportive care needs, a linear mixed effects model was used with repeated measures using restricted maximum likelihood and an unstructured covariance type, which takes into account intraparticipant correlations of outcomes and allows for the introduction of time-varying covariates. The results report regression estimates and p values for fixed effects, along with the Akaike information criterion (AIC); the smaller the AIC is, the better the model fit. Using a stepwise procedure, three models were constructed. First, in the basic model (Model 1), Type D personality and demographic and clinical variables (e.g. age, educational status, pathological stage, type of surgery, comorbidities, and adjuvant therapy) were entered. In step 2, we adjusted the model for symptom burden (e.g. number of symptoms, severity of symptoms, and degree of disruption to life). Finally, social support, which may contribute to the need for supportive care, was included in Model 3. We chose models with the lowest goodness-of-fit statistics (AIC). All analyses were conducted using the statistical software IBM SPSS Statistics 24.0. Tests were two-tailed, with a P value < 0.05 indicating statistical significance.

## Results

### Sample characteristics

**Fig. 1:**
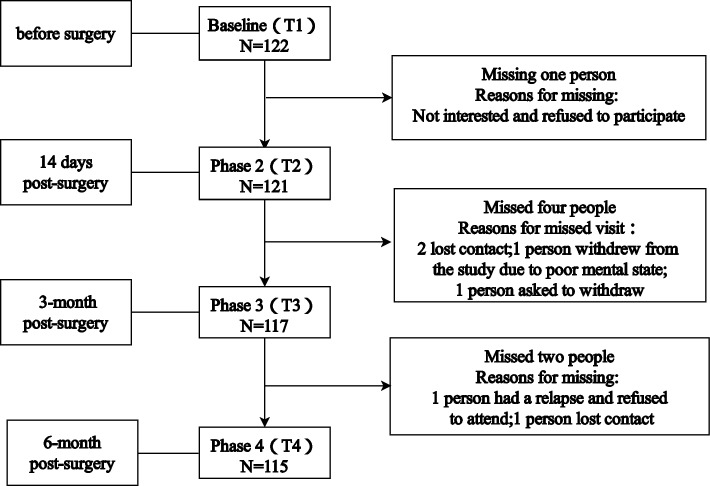
Flow chart of participant follow-up completion

A total of 122 patients were studied, of which 122 valid questionnaires were collected at baseline. One patient was missed at the 14-day postsurgery (T2) medication change follow-up, 4 were missed at the 3-month postsurgery (T3) follow-up, and 2 were missed at the 6-month postsurgery (T4) follow-up. The response rate was 94.26%. The sample flow is shown in Fig. 1. Elderly patients who refused or were lost to follow-up did not differ by demographic or medical factors or by their responses to the study variables measured at baseline. Among the 122 elderly patients, all were female, and the age ranged from 65 to 84 years, with an average of 69.51 ± 4.56 years. The participants mainly had a junior high school education level (*n* = 32, 26.2%) and were married (*n* = 96, 78.7%). More than 70% of the participants had one (*n* = 55, 45.0%) or more (*n* = 35, 28.6%) comorbidities, and their pathological stage was mostly concentrated in stages I (*n* = 38, 31.1%) and II (*n* = 52, 42.6%). Regarding adjuvant therapy, 27 (22.6%) patients underwent surgery only, 52 (42.1%) underwent surgery with chemotherapy, and 42 (35.2%) underwent surgery with other therapies. Table 1 summarizes the demographic and clinical characteristics of these participants.

**Table 1:** Baseline demographic and clinical characteristics of all patients

Variables	*n* (%) /mean ± SD
Age at baseline (years)	69.51 ± 4.56
Marital status	
Married	96(78.7)
Single, divorced or widowed	26(21.3)
Educational status	
Primary school	48(39.4)
Middle school	32(26.2)
High school	33(27.0)
College or university	9(7.4)
Geographic location	
Countryside	30(24.6)
Metropolitan	92(75.4)
Primary Caregiver	
Spouse	55(45.1)
Children	35(28.7)
Others or None	32(26.2)
Number of children	
None	3(2.40)
1	91(74.5)
≥ 2	28(23.1)
Family history of the breast cancer	
Yes	4(3.2)
No	118(96.7)
Comorbidities	
None	32(26.2)
1	55(45.0)
> 1	35(28.6)
Type of surgery	
Breast-conserving therapy	38(31.1)
Modified radical mastectomy	84(68.9)
Pathological stage	
I	38(31.1)
II	52(42.6)
III	32(26.2)
Adjuvant therapy	
Surgery only	27(22.6)
Surgery + Chemotherapy	52(42.1)
Surgery + others	43(35.2)

### Between-group differences in supportive care needs at different time points

**Fig. 2:**
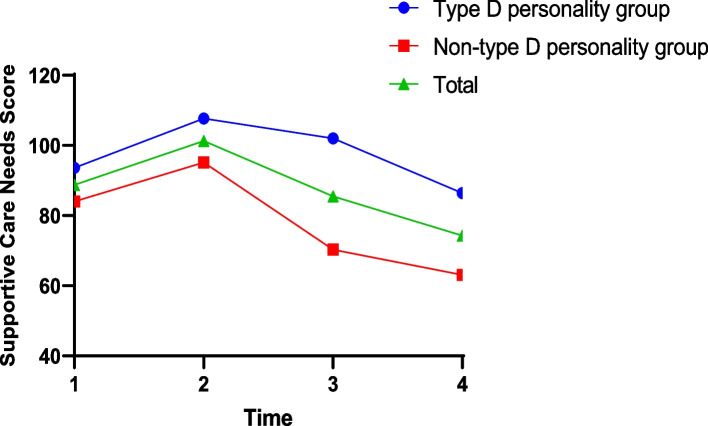
Supportive care needs scores over time stratified by Type D personality

Figure 2 shows that the supportive care needs of the Type D personality group, non-Type D personality group, and the population as a whole tended to increase from presurgery (T1) to 14 days postsurgery (T2), after which the level of supportive care needs showed a decreasing trend. The level of need was consistently higher in the Type D personality group than in the non-Type D personality group. Further analysis of the differences between the Type D and non-Type D personality groups was conducted by t tests, shown in Table 2, which indicated statistically significant differences between the two groups at the four time points. The slope obtained from the multivariable, linear mixed-effects model analysis demonstrated that the supportive care needs scores of elderly breast cancer patients significantly declined during the whole study period (β =  − 5.06, *p* < 0.001). The rate of decline was lower in the Type D personality group than in the non-Type D personality group, with a longer overall duration (β = 4.20, *p* = 0.001).

**Table 2:** Student's t test for Type D and Non-Type D personality in domains of supportive care needs at different time points

Time	Personality(n)	Supportive care needs	t	*P*
T1	Non-Type D (62)	84.00	-4.063	< 0.001
	Type D (60)	93.65		
T2	Non-Type D (62)	95.19	-5.905	< 0.001
	Type D (59)	107.69		
T3	Non-Type D (61)	70.33	-10.929	< 0.001
	Type D (56)	102.05		
T4	Non-Type D (60)	63.10	-9.561	< 0.001
	Type D (55)	86.45		

### Effects of Type D personality and related factors on changes in the supportive care needs of older breast cancer patients over time

Type D personality and an interaction term between Type D personality and time were entered in the model to assess the between-subject and within-subject effects of Type D personality on the supportive care needs of elderly breast cancer patients. Table 3 shows the results obtained from the multivariable, linear mixed-effects model analysis to identify factors associated with changes in supportive care need scores over time in elderly breast cancer patients. Using a stepwise procedure, three models were calculated. We entered the Type D personality, demographic and clinical information variables in the first step. The results showed a significant between-subjects effect (β = 4.22, p = 0.014) and within-subjects impact on Type D personality (β = 3.59, *p* = 0.001). Older patients had lower levels of supportive care needs (β = -0.72, *p* < 0.001). Patients with higher education levels had higher levels of supportive care needs. In addition, patients whose pathological stage was stage I had lower levels of supportive care needs than those whose pathological stage was stage III (β = -0.05, *p* = 0.025). The more comorbidities patients had, the higher their supportive care needs were (β = -5.70, *p* = 0.004; β = -4.32, *p* = 0.010). Elderly patients undergoing surgery and chemotherapy or others had higher supportive care needs than those undergoing surgery only (β = 6.12,* p* = 0.001; β = 5.28,* p* = 0.008). After adjusting for symptom burden covariates in Step 2, the results showed a significant difference in the within-subjects effect of Type D personality (β = 4.16, *p* = 0.002) but no significant between-subjects effect was shown (β = 2.80, *p* = 0.192). The supportive care needs of patients with lower education levels (primary or middle school) were higher than those of patients with a tertiary education and above (β = -16.47, *p* < 0.001; β = -7.17, *p* = 0.025). Patients with more comorbidities had relatively higher supportive care needs (β = -4.93, *p* = 0.023, β = -3.31, *p* = 0.044). Receiving surgery and chemotherapy or other adjuvant therapy had more substantial effects on the supportive care needs of elderly patients (β = 10.46,* p* < 0.001; β = 9.23, *p* < 0.001), which was similar to Model 1. The higher the degree of the impact of symptoms on life was, the higher the supportive care needs of older patients (β = 0.20, *p* = 0.006). In the final step, we included the social support variable. The relationship between Type D personality and supportive care needs was similar to that in Model 2. Age, educational status, pathological stage, comorbidities, adjuvant therapy, severity of symptoms, and degree of disruption to life were strongly related to supportive care needs. Although social support did not reach a significant level, the model's AIC values decreased significantly with the inclusion of social support. With the changes in the AIC value, a reduction in almost all outcomes was identified, especially in Model 2; Model 3 had the smallest AIC value with the inclusion of social support variables, which was the optimal model.

**Table 3:** Factors associated with changes in supportive care needs over time

	Model 1		Model 2		Model 3	
	β	*P*	β	*P*	β	*P*
Time	-6.91	< 0.001	-12.80	< 0.001	-13.20	< 0.001
Personality between	4.22	0.014	2.80	0.192	3.61	0.10
Personality within	3.59	0.001	4.16	0.002	4.37	0.005
Age	-0.72	< 0.001	-0.80	< 0.001	-0.82	< 0.001
Educational status(College or university)	Reference					
Primary school	-15.41	< 0.001	-16.47	< 0.001	-16.43	< 0.001
Middle school	-6.90	0.016	-7.17	0.025	-6.88	0.047
High school	-4.32	0.130	-4.94	0.114	-5.14	0.129
Pathological stage (III)	Reference					
I	-0.05	0.025	-2.13	0.304	-3.31	0.142
II	-1.77	0.313	-4.48	0.025	-5.19	0.018
Type of surgery (Modified radical mastectomy)	Reference					
Type of surgery (Breast conserving therapy)	1.42	0.332	0.02	0.992	-0.33	0.853
Comorbidities (> 1)	reference					
Comorbidities (None)	-5.70	0.004	-4.93	0.023	-5.22	0.028
Comorbidities (1)	-4.32	0.010	-3.31	0.044	-3.393	0.042
Adjuvant therapy (Surgery only)	reference					
Adjuvant therapy (Surgery + Chemotherapy)	6.12	0.001	10.46	< 0.001	11.09	< 0.001
Adjuvant therapy (Surgery + others)	5.28	0.008	9.23	< 0.001	9.32	< 0.001
Number of symptoms			0.04	0.876	0.01	0.993
Severity of symptoms			0.03	0.594	0.04	0.043
Degree of disruption to life			0.20	0.006	0.18	0.017
Social support					0.13	0.400
Akaike information criterion (AIC)	3849.644		2830.972		2819.751	

Model 1: Age, educational status, pathological stage, type of surgery, comorbidities, adjuvant therapy

Model 2: Model 1 + number of symptoms, severity of symptoms, degree of disruption to life

Model 3: Model 2 + social support

## Discussion

To the best of our knowledge, there has been no study on Type D personality and other factors associated with longitudinal changes in the supportive care needs of elderly breast cancer survivors. It is one of the first longitudinal studies among elderly breast cancer patients to analyse the impact of Type D personality on supportive care needs over six months after surgery. This study showed that in the elderly breast cancer patient population, there was an overall decreasing trend in supportive care needs. Patients with Type D personality were more likely to have higher scores for supportive care needs at each stage than those with non-Type D personality. Additionally, demographic and clinical variables (e.g., age, educational status, pathological stage, comorbidities, and adjuvant therapy), symptom burden, and Type D personality were found to significantly influence supportive care needs among elderly breast cancer patients in this study, which indicates that Type D personality is an essential factor influencing the needs of elderly patients and requires focused attention.

### The supportive care needs of elderly breast cancer patients change dynamically

This study found that supportive care needs of elderly breast cancer patients increased to a maximum at 14 days postoperatively (T2) and gradually decreased thereafter, which is similar to Zhang's study [21]. It was found that the needs of breast cancer patients culminated at 14 days postsurgery (T2) due to drains and painful postoperative incisions, which led to upper limb dysfunction and severely affected the patients’ physiology and life [21]. Thereafter, the supportive care needs of elderly breast cancer patients gradually declined over time. However, it is worth noting that at 6 months postoperatively, the patients' supportive care need scores were still high, which suggests that the effect of time on need changes is limited, thus further exploration of the factors affecting supportive care needs of elderly patients is necessary.

### Type D personality and factors influencing supportive care needs

Our study found that demographics such as age and educational status significantly influenced the supportive care needs of elderly breast cancer patients. Older patients had relatively lower levels of supportive care needs, which is consistent with the findings of Li [22]. Li et al. found that younger patients may have more needs regarding body image, sexuality and relationships [22]. Our study also found that patients with higher education levels had higher supportive care needs. Similar results were found in Cheah's research [38]. In addition, this study also found that clinical data, such as comorbidities, pathological stage, and adjuvant therapy, significantly influenced supportive care needs. Increased comorbidities and pathological stages often make it more difficult to treat the disease, resulting in high supportive care needs. Of considerable interest is that this study found that patients receiving subsequent chemotherapy or other treatments had higher supportive care needs than those treated with surgery only. Studies have found that treatments such as chemotherapy and radiotherapy are often accompanied by a series of adverse reactions, which are more severe for patients, and that elderly cancer patients are relatively less resilient to adverse reactions and therefore have higher levels of unmet supportive care needs [39, 40].

The degree of symptom interference and the severity of symptoms in patients' lives also significantly influenced changes in the needs of elderly breast cancer patients. Ren [41] also found that patients with higher symptom burden had higher levels of supportive care needs. This may lead to an increased level of supportive care needs for elderly breast cancer patients, possibly because they often experience adverse symptoms with treatment, such as pain, fatigue, nausea, and sleep disturbances. The complete model showed that the social support score did not significantly influence the change in patients' needs, which is different from the study by Lou [42], although the addition of social support optimized the model. This may be due to differences in the data collection period of the study.

There was a difference in the level of and change in supportive care needs between the Type D and non-Type D personality groups. This difference persisted after controlling for demographic and clinical information, symptom burden and social support. Therefore, there is a strong need to focus on the impact of patients' personality traits on their supportive care needs to facilitate the development of individually tailored interventions by health care professionals. Shun et al. [43] also found relatively high levels of supportive care needs in patients with Type D personality. In addition, some studies have found that Type D personality not only directly influences patients' needs but also indirectly influences their supportive care needs through the mediation of factors such as social support and coping styles [44, 45]. However, research on the effect of Type D personality on changes in patients' supportive care needs is currently lacking, and comparability between the available findings and our study is limited because of differences in the study type, study population, and follow-up time. Future research should strengthen the focus on the supportive care needs of elderly breast cancer patients and further explore supportive care needs in association with Type D personality and other related factors.

### Study strengths and limitations

The strengths of our study include (a) the focus on the elderly breast cancer population in China and (b) the longitudinal design used to analyse the impact of Type D personality on supportive care needs. Despite these strengths, the following must be considered when interpreting the results: (a) only Type D personality was assessed, ignoring the impact of other personality traits on supportive care needs;(b) this study focused only on breast cancer patients. Thus, future studies should cover elderly patient with different types of cancer and explore the impact of other personality traits, such as Type A personality, on patients' supportive care needs; and (c) this study was conducted during the COVID-19 period, when patients' social support was generally lower, and no significant association between social support and supportive care needs was found in this study. Therefore, further studies are needed to explore the relationship between social support and supportive care needs.

### Clinical implications

Type D personality is an important factor influencing the supportive care needs of elderly breast cancer patients. Health care providers need to focus on supportive care for elderly breast cancer patients with Type D personality. Further study is needed to better understand unmet care needs based on personality traits and to develop individually tailored intervention programs.

## Conclusion

Our study found that Type D personality plays a vital role in the change in supportive care needs in elderly breast cancer patients. Elderly breast cancer patients with Type D personality had significantly higher levels of supportive care needs than patient with non-Type D personality. These findings suggest that clinical care providers need to pay more attention to the supportive care needs of elderly breast cancer patients with Type D personality and can provide helpful information for appropriate interventions and strategies for changes in supportive care needs in patients with different personality traits. Future work is needed to better understand unmet care needs based on personality traits and to develop individualized interventions.

## Data Availability

The datasets used and analyzed during the present study are available from the corresponding author at the reasonable request.
